# The Evolution and Impact of Artificial Intelligence in Orthognathic Surgery: A Bibliometric Analysis

**DOI:** 10.7759/cureus.115442

**Published:** 2026-08-30

**Authors:** Sonia Naija, Anis Chaouch

**Affiliations:** 1 Oral and Maxillofacial Surgery, Principal Military Hospital of Instruction of Tunis (HMPIT), Tunis, TUN; 2 Telecommunications Engineering, Università di Pisa, Pisa, ITA

**Keywords:** artificial intelligence, bibliometric analysis, cephalometry, deep learning, generative ai, maxillofacial surgery, orthognathic surgery, virtual surgical planning

## Abstract

Artificial intelligence (AI), machine learning (ML), and deep learning (DL) are reshaping orthognathic surgery and maxillofacial traumatology by supporting diagnostic imaging and three-dimensional virtual surgical planning (VSP). This study provides a bibliometric mapping of the field in order to characterise its thematic structure, trace its temporal evolution, describe international collaboration patterns, and situate the clinical limitations reported in the literature. A systematic search was conducted in Scopus, PubMed/MEDLINE, and IEEE Xplore from database inception to 24 August 2026, restricted to English-language records. The query combined three blocks: artificial intelligence terminology ("artificial intelligence", "machine learning", "deep learning", "neural network*", "machine intelligence"), craniomaxillofacial terms ("orthognathic", "maxillofacial", "mandibular split", "le fort", "stomatognathic"), and surgical terms ("surgery", "surgical", "planning", "osteotomy"). Records were eligible when they both implemented an AI method and applied it to the craniomaxillofacial or dental domain, including educational and patient-communication applications; records were excluded as editorials, errata, letters and short opinion pieces, conference proceedings volumes, studies without an AI method, medical head-and-neck oncology, three-dimensional facial reconstruction without craniomaxillofacial surgical application, non-clinical computer vision, or topics outside the craniomaxillofacial domain. The search retrieved 1,925 records; after removal of 539 duplicates, 1,386 unique records underwent title and abstract screening, and 854 documents met the eligibility criteria. Bibliometric networks were constructed in VOSviewer (version 1.6.21; Centre for Science and Technology Studies (CWTS), Leiden University, Netherlands). Keyword co-occurrence analysis resolved five thematic clusters, centred respectively on generative AI and language models, orthognathic surgery and cephalometry, dentomaxillary radiological imaging, DL methodology, and computer-assisted surgery with digital dentistry. Temporal overlay analysis showed that keywords relating to large language models and educational applications carry the most recent average publication years, whereas cephalometric and landmark detection terms are comparatively older. Country-level co-authorship analysis identified 35 countries meeting a five-document threshold, organised into six regional clusters, with China and the United States as the principal contributors by output and the United States as the most central connector. These bibliometric observations indicate a field in rapid thematic transition, in which methodological heterogeneity, dataset homogeneity, and limited algorithmic interpretability remain the barriers most frequently reported to clinical translation.

## Introduction and background

Artificial intelligence (AI), machine learning (ML), and deep learning (DL) are transforming the clinical landscape of orthognathic surgery and oral-maxillofacial traumatology [1,2]. Conventional orthognathic planning relied on two-dimensional lateral cephalometric tracing, plaster model surgery, and manually fabricated acrylic splints. This workflow constrained the assessment of transverse discrepancies and facial asymmetry, both of which are poorly represented in a single lateral projection and made the predicted outcome dependent on the operator's manual reproduction of the plan. The introduction of three-dimensional virtual surgical planning (VSP), based on cone-beam computed tomography and surface scanning, allowed skeletal movements to be simulated volumetrically and transferred to the operating room through computer-designed guides and patient-specific plates, establishing the anatomical accuracy and predictability on which subsequent algorithmic automation now depends [3]. AI was therefore not grafted onto an analogue workflow but onto an already digital one, in which anatomical structures were segmented, landmarks defined and surgical movements parameterised - that is, onto data that a learning algorithm can consume. Digital tools are increasingly deployed to automate radiographic diagnosis, enhance VSP, predict treatment outcomes and streamline patient workflows. Convolutional neural networks (CNNs) and transformer-based architectures are widely investigated to automate cephalometric landmark detection, perform skeletal segmentation, and assist in designing patient-specific osteosynthesis plates [4]. Despite these advances, routine clinical integration remains constrained by methodological inconsistency, training dataset limitations, and unresolved ethical and legal questions.

Bibliometric analysis is a quantitative methodology for mapping scientific output, identifying research hotspots and tracing collaboration structures. Several narrative and scoping reviews have described AI applications in this specialty [1,2,5], but they characterise the field qualitatively and do not quantify its thematic structure or its collaboration networks.

Bibliometric analyses of AI in this specialty have also begun to appear, mapping research in oral and maxillofacial surgery [6,7], in orthodontics and orthognathic surgery [8,9], and in maxillofacial skeletal reconstruction [10]. These studies have relied on Scopus and Web of Science, either individually or in combination, and one restricted its analysis to the 100 most-cited articles [8]. None has incorporated IEEE Xplore, although a substantial part of the methodological work on craniofacial DL is published in the engineering literature; in the present corpus, 108 included documents were retrievable only from that database. Existing analyses have also reported limited detail on eligibility criteria, screening agreement and terminological standardisation.

The present study therefore differs in three respects: it pools three databases spanning both the biomedical and the engineering literature, it reports explicit eligibility criteria together with a measure of screening agreement and a categorised exclusion log, and it applies and publishes a terminological thesaurus so that the keyword networks can be reproduced exactly. It further extends coverage to August 2026, capturing the most recent generative-AI research front.

## Review

Search strategy and bibliometric construction

Three databases were interrogated: Scopus, PubMed/MEDLINE, and IEEE Xplore. Multiple databases were used in order to capture both the biomedical and the engineering literature on this topic, which are only partially co-indexed. The three queries interrogate the same conceptual space, structured in three blocks (AI terminology × craniomaxillofacial domain × surgical act), and are reproducible as stated. Search parameters are detailed in Table 1.

**Table 1:** Database search strategy and parameters for literature extraction.

Parameter	Specification
Databases	Scopus (Elsevier), PubMed/MEDLINE, IEEE Xplore
Query field	Scopus: TITLE-ABS-KEY; PubMed: [tiab]; IEEE Xplore: All Metadata
Boolean structure	( "artificial intelligence" OR "machine learning" OR "deep learning" OR "neural network*" OR "machine intelligence" ) AND ( "orthognathic" OR "maxillofacial" OR "mandibular split" OR "le fort" OR "stomatognathic" ) AND ( "surgery" OR "surgical" OR "planning" OR "osteotomy" )
Language filter	English only (Scopus: LANGUAGE(english); PubMed: English[lang])
Temporal coverage	Inception to 24 August 2026
Records retrieved	Scopus 854; PubMed 524; IEEE Xplore 547; total 1,925

Eligibility criteria

Records were included when they met both of the following conditions: (i) implementation of an AI method (AI, ML, DL, neural networks, CNNs, transformers, large language models, random forests, support vector machines, gradient boosting, U-Net architectures, radiomics or reinforcement learning); and (ii) application to the craniomaxillofacial or dental domain, including educational, academic and patient-communication applications within the specialty.

Records were excluded for any of the following reasons: editorials, errata, letters to the editor, commentaries, and short opinion pieces without primary data; conference proceedings volumes, as distinct from individual papers published within such proceedings, which were retained; absence of an AI method; medical head-and-neck oncology (prognosis, immunotherapy, molecular biomarkers); three-dimensional facial reconstruction tasks lacking direct craniomaxillofacial surgical application; non-clinical computer vision; and topics outside the craniomaxillofacial domain.

Screening procedure

Title and abstract screening was performed using two independent AI-assisted screening passes against the predefined criteria, conducted with two different large language model systems (Claude, Anthropic PBC, San Francisco, CA; Gemini, Google, Mountain View, CA). Agreement between the two passes gave a raw agreement of 79.9% and a Cohen's kappa of 0.33. All 83 discrepant records were subsequently adjudicated independently by the two authors (SN, AC) against the stated eligibility criteria, with disagreements resolved by discussion. A final consistency audit of all screening decisions against the definitive criteria was performed prior to analysis.

The search retrieved 1,925 records (Scopus: 854, PubMed: 524, and IEEE Xplore: 547). After removal of 539 duplicates by DOI and by normalised title and year, 1,386 unique records were screened. A total of 532 records were excluded: absence of an AI method (n = 202), non-clinical computer vision (n = 185), outside the craniomaxillofacial domain (n = 91), editorials and opinion letters (n = 19), three-dimensional facial reconstruction without craniomaxillofacial application (n = 11), medical head-and-neck oncology (n = 16), and conference proceedings volumes (n = 8). The final corpus comprised 854 documents (712 journal articles, 131 conference papers, 11 book chapters). The selection process is reported following the PRISMA 2020 statement [11], adapted to a bibliometric workflow: record counts and exclusion reasons are reported at each stage, whereas items relating to the appraisal of clinical evidence are not applicable to this design. This selection process is summarised in Figure 1.

**Figure 1:**
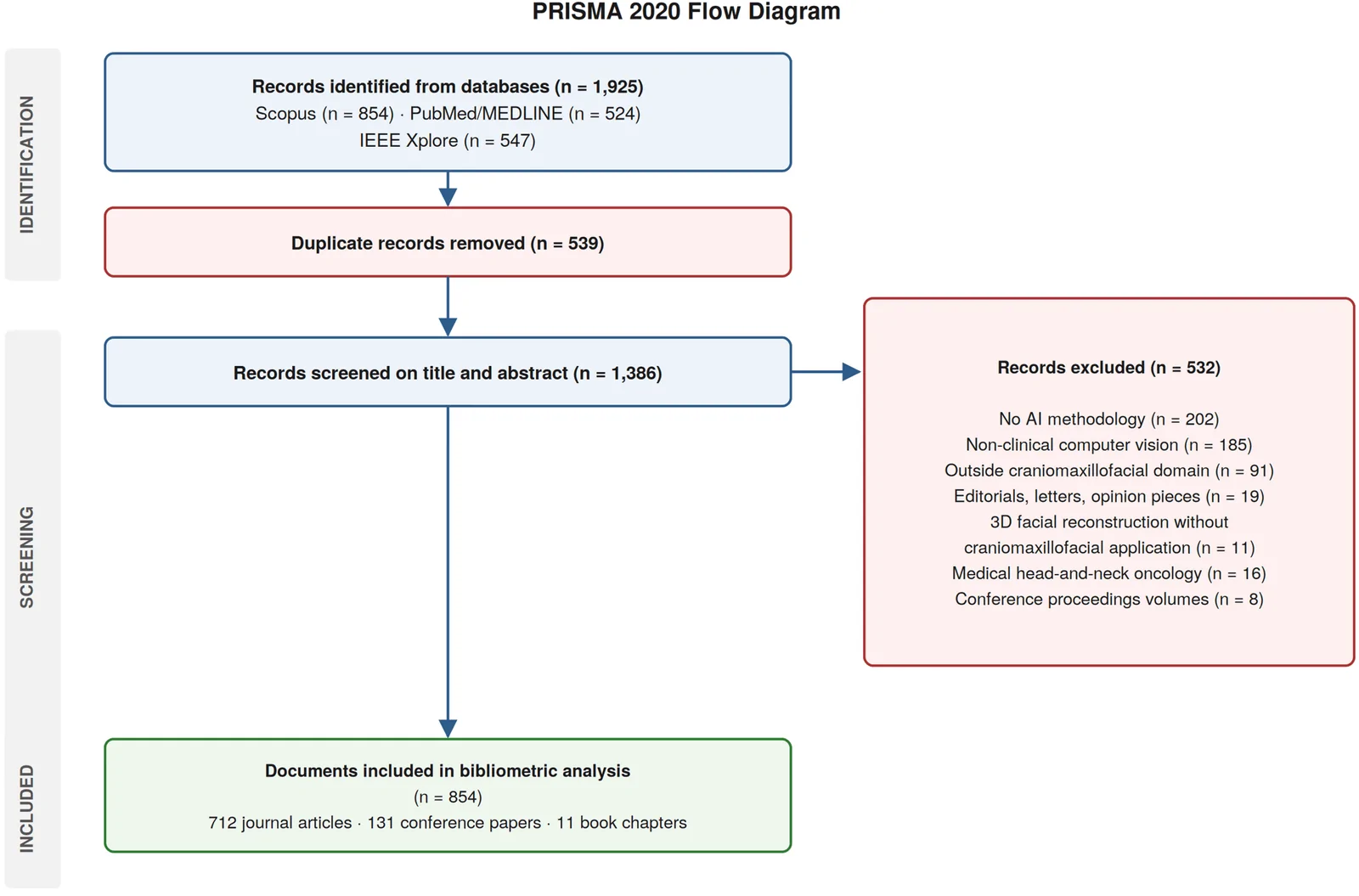
PRISMA 2020 flow diagram of the study selection process. Records were identified from three databases (Scopus, PubMed/MEDLINE, and IEEE Xplore) from inception to 24 August 2026, restricted to English-language publications. After removal of 539 duplicates by digital object identifier and by normalised title with year of publication, 1,386 unique records underwent title and abstract screening. Screening was performed using two independent AI-assisted passes against predefined eligibility criteria (raw agreement: 79.9%; Cohen's κ = 0.33), and all 83 discrepant records were adjudicated by the authors. A total of 532 records were excluded, and 854 documents were included in the bibliometric analysis.

Data extraction

For each included record, the following metadata were extracted from the database exports: title, authors, publication year, source title, digital object identifier, author and index keywords, abstract, and author affiliations. Extraction was performed programmatically from the exported files rather than manually, so that the dataset underlying the networks corresponds exactly to the database records; the resulting dataset is deposited on Zenodo (doi:10.5281/zenodo.22131818). No study-level risk-of-bias instrument was applied, as the unit of analysis is the bibliographic record and its metadata rather than the clinical findings of individual studies, and no effect estimates are extracted or synthesised.

Bibliometric analysis

Networks were constructed in VOSviewer (version 1.6.21; Centre for Science and Technology Studies (CWTS), Leiden University, Netherlands). Author keyword co-occurrence networks (Figures 2-3) were built from the pooled corpus using the full counting method. A custom VOSviewer thesaurus file was applied to remove generic database indexing terms, such as population descriptors and publication-type labels, and to merge orthographic and case variants of identical concepts; this file is deposited alongside the corpus dataset on Zenodo. A minimum occurrence threshold of 10 was applied, and 124 of 4,130 author keywords met this threshold. The resulting network comprised 124 items in five clusters, connected by 4,105 links with a total link strength of 20,261.

**Figure 2:**
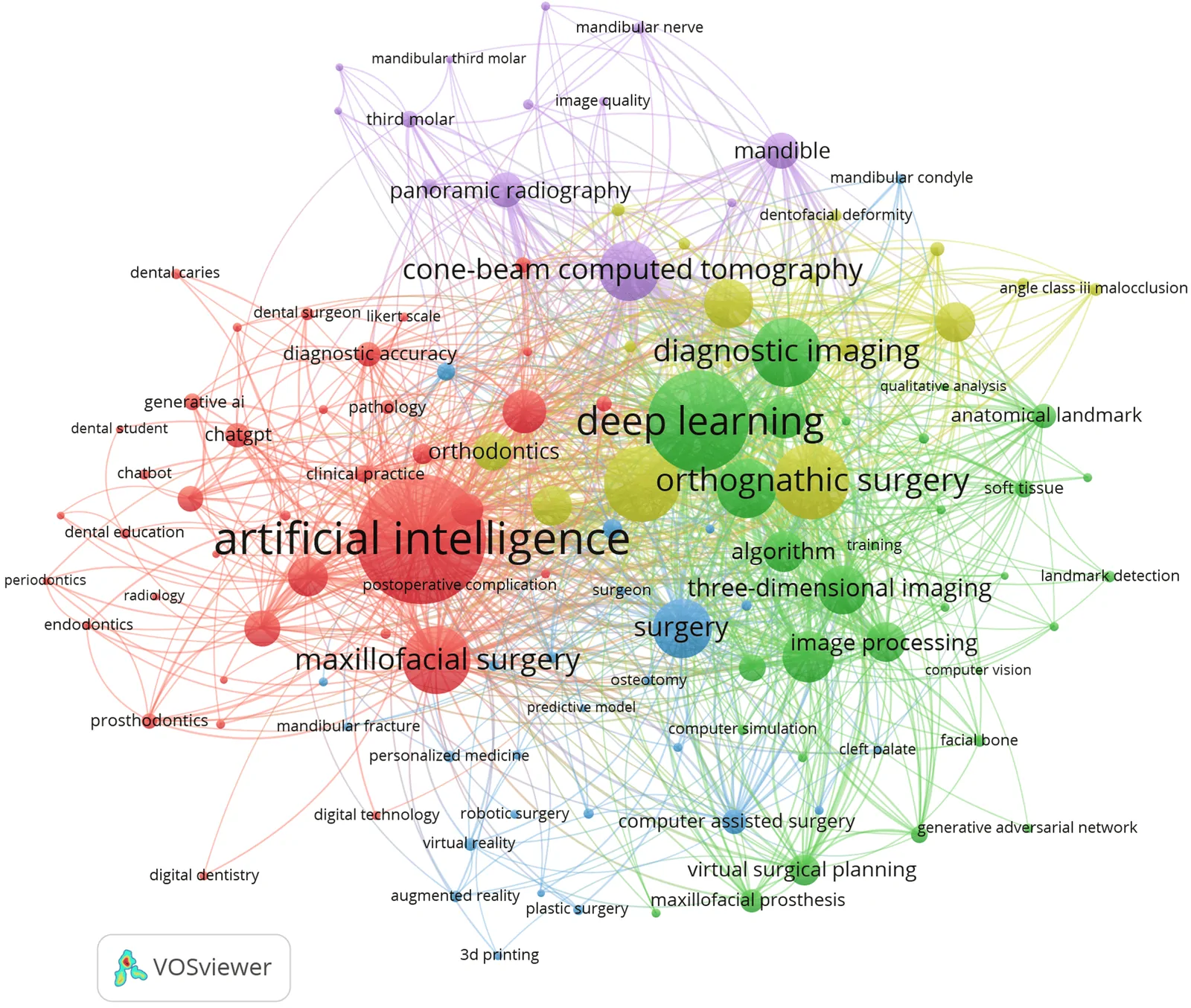
VOSviewer network visualisation of author keyword co-occurrence in artificial intelligence research in orthognathic and maxillofacial surgery. Network constructed from 854 documents using VOSviewer (version 1.6.21; Centre for Science and Technology Studies (CWTS), Leiden University, Netherlands), with the full counting method and a custom thesaurus file removing generic database indexing terms and merging orthographic variants. A minimum occurrence threshold of 10 was applied, and 124 of 4,130 author keywords met this threshold. Node size reflects keyword occurrence frequency, line thickness indicates co-occurrence link strength, and colours denote the five clusters identified by the VOS clustering technique. The network comprises 124 items connected by 4,105 links, with a total link strength of 20,261.

**Figure 3:**
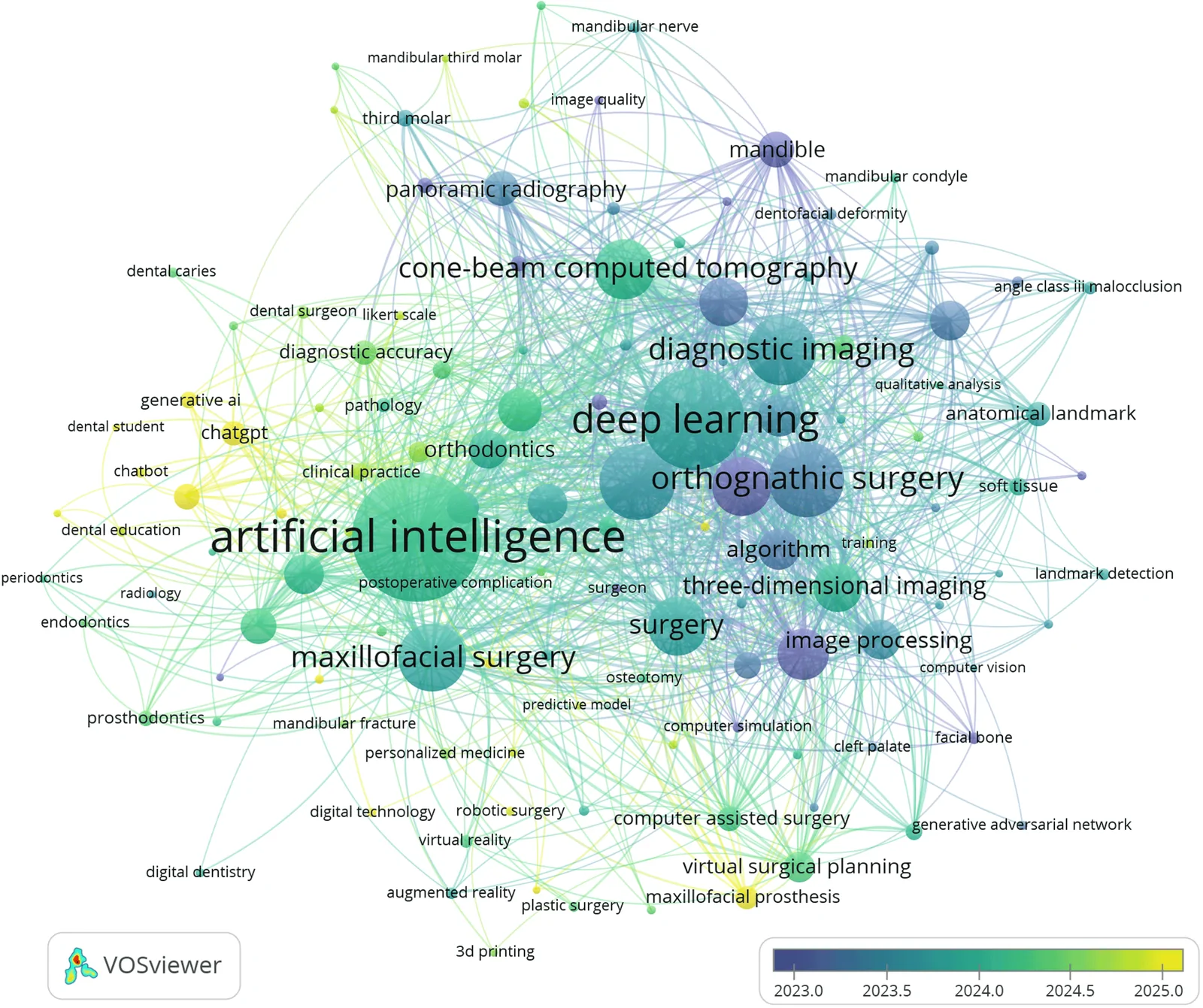
Temporal overlay visualisation of author keyword co-occurrence (average publication year). The same network as Figure 2, with node colour determined by the average publication year of the documents in which each keyword occurs, ranging from 2023.0 (dark blue) to 2025.0 (yellow). Keywords with the most recent average publication years - including ChatGPT, large language model, chatbot, dental education, and educational measurement - indicate the emerging thematic front of the field, whereas cephalometry, orthognathic surgery, feature extraction, and training represent comparatively established lines of research.

The international co-authorship network (Figure 4) was constructed from the Scopus subset of the corpus, exported on 25 August 2026 (n = 855 records), because complete author affiliation data required for country-level aggregation were available only from this database. Countries were used as the unit of analysis with full counting, and documents co-authored by more than 25 countries were excluded. A minimum threshold of five documents per country was applied; of 103 countries identified, 35 were retained, forming 6 clusters connected by 217 links with a total link strength of 543. In all analyses, link strengths were normalised using the association strength method, and clustering was performed with a resolution parameter of 1.00 and a minimum cluster size of 1. For display purposes, the minimum link strength for drawn lines was set to 2.00 for the keyword co-occurrence networks (Figures 2-3) and left at 0 for the country co-authorship network (Figure 4), reflecting the differing density of the two networks; the maximum number of displayed links was left at the VOSviewer default of 1,000 in both cases. These settings govern visual rendering only and do not affect the clustering solution.

**Figure 4:**
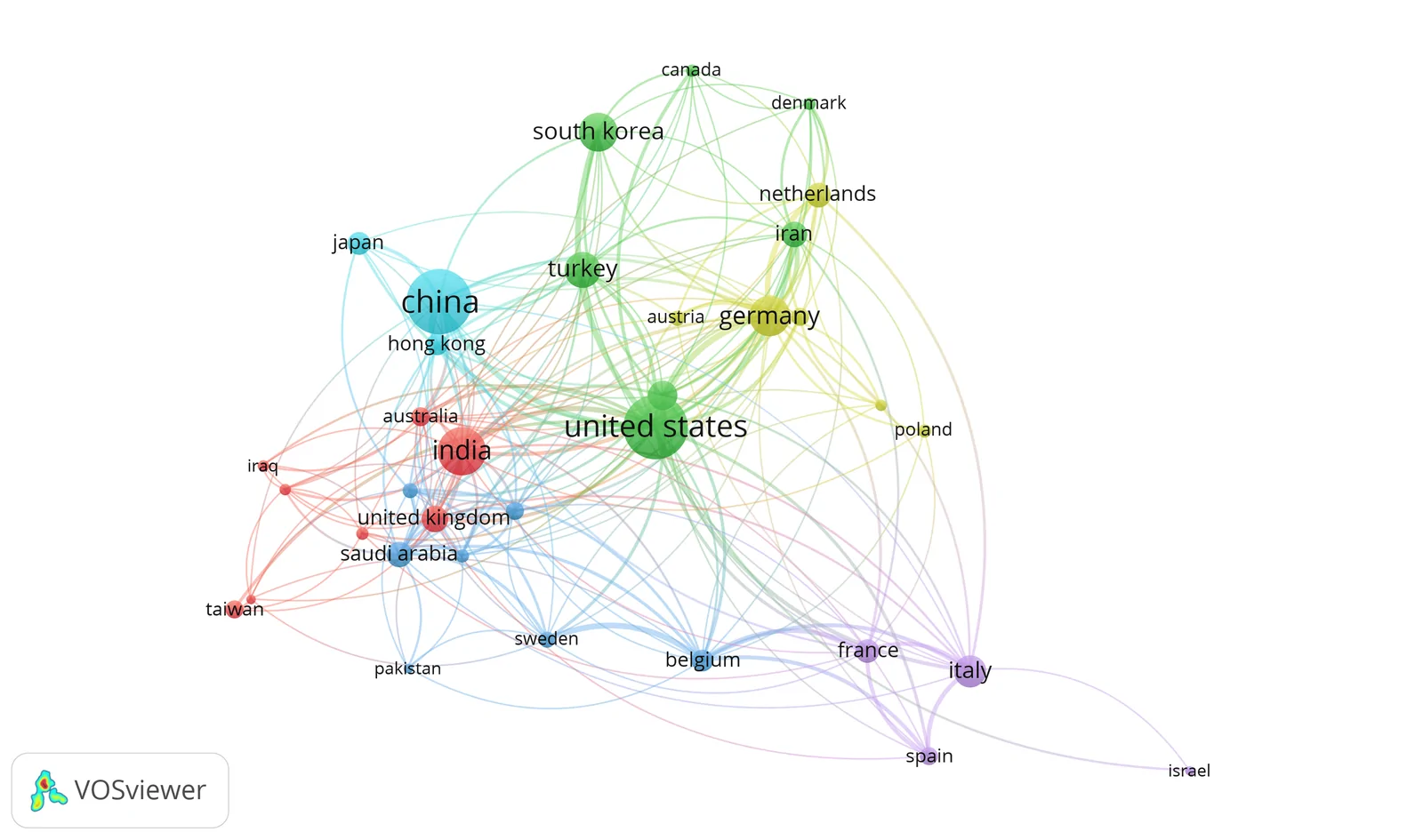
VOSviewer network visualisation of international co-authorship. Network constructed from the Scopus subset of the corpus (n = 855 records; extraction date: 25 August 2026), as complete author affiliation data required for country-level aggregation were available only from this database. Countries were used as the unit of analysis with the full counting method, and documents co-authored by more than 25 countries were excluded. A minimum threshold of five documents per country was applied; of 103 countries identified, 35 were retained. Node size reflects the number of documents per country, line thickness indicates co-authorship link strength, and colours denote clusters of preferentially collaborating countries. The network comprises 35 items in six clusters, connected by 217 links with a total link strength of 543.

Thematic domains and research hotspots

The co-occurrence network of author keywords (Figure 2) comprised 124 keywords connected by 4,105 links with a total link strength of 20,261, resolved into five thematic clusters summarised in Table 2. The network is organised around three high-frequency hubs - AI, DL, and orthognathic surgery - reflecting the dual nature of the field, in which methodological development and clinical application are closely intertwined.

**Table 2:** Summary of VOSviewer bibliometric clusters and corresponding clinical domains.

Cluster	Principal keywords	Clinical domain
1 (red)	artificial intelligence, chatgpt, large language model, chatbot, natural language processing, dental education, educational measurement, clinical decision support, diagnosis, prosthodontics	Generative AI, large language models and educational applications: clinical decision support, practitioner and student training, patient communication.
2 (green)	deep learning, computed tomography, three-dimensional imaging, neural network, diagnostic imaging, segmentation, image processing, algorithm, feature extraction, generative adversarial network	Deep learning and computer vision: network architectures, three-dimensional and diagnostic imaging, segmentation, image generation, virtual surgical planning.
3 (purple)	cone-beam computed tomography, panoramic radiography, mandible, mandibular canal, mandibular nerve, third molar, tooth extraction, tooth impaction, image quality	Dentomaxillary radiological imaging: segmentation and anatomical landmarking on CBCT and panoramic radiographs, pre-extraction risk assessment.
4 (yellow)	orthognathic surgery, cephalometry, machine learning, orthodontics, maxilla, malocclusion, angle class III malocclusion, treatment planning, orthodontic procedure, convolutional neural network	Orthognathic surgery and cephalometric analysis: machine learning/CNN-based treatment planning, orthodontic diagnosis and malocclusion classification.
5 (blue)	surgery, computer assisted surgery, virtual reality, augmented reality, temporomandibular joint, treatment outcome, plastic surgery, 3d printing, personalized medicine, magnetic resonance imaging	Computer-assisted and reconstructive surgery: virtual/augmented reality planning, treatment outcome assessment, temporomandibular joint disorders, personalized 3D-printed solutions.

Cluster 1 groups generative AI and large language model applications with educational and decision-support keywords, indicating that conversational systems in this specialty are currently investigated primarily for training, patient communication, and clinical decision support rather than for operative planning. Cluster 2 assembles the methodological vocabulary of DL and diagnostic imaging - network training, feature extraction, segmentation, and generative adversarial networks - applied to three-dimensional and virtual surgical planning. Cluster 3 centres on radiological imaging of the mandible and dentition, where automated segmentation of the mandibular canal and third molar risk assessment are prominent. Cluster 4 corresponds to the core orthognathic domain, associating cephalometric analysis, ML, and CNN methods with orthodontic treatment planning and malocclusion classification. Beyond static landmark identification, AI-driven architectures in this domain are increasingly applied to longitudinal cephalometric superimposition in order to characterise craniofacial growth over time [12]. Cluster 5 links computer-assisted and reconstructive surgery with virtual and augmented reality planning, treatment outcome assessment, and temporomandibular joint disorders.

Temporal evolution and global collaboration

The temporal overlay visualisation (Figure 3) maps the average publication year onto the same network, with values ranging from 2023.0 to 2025.0. Keywords with the most recent average publication years include chatgpt, large language model, chatbot, dental education, and educational measurement, whereas cephalometry, orthognathic surgery, feature extraction, and training carry earlier average years. This gradient indicates that generative language applications constitute the most recent thematic front in this literature, while cephalometric automation represents a comparatively established line of work.

The distribution of the corpus by year of publication supports this reading: annual output remained below 25 documents until 2020, rose to 51 in 2021, 77 in 2022, and 90 in 2023; and then accelerated to 161 in 2024 and 226 in 2025. The year 2026, incomplete at the time of extraction, already accounts for 186 documents.

The international co-authorship network (Figure 4) identified 103 contributing countries, of which 35 met the threshold of at least five documents. China (163 documents) and the United States (155) are the leading contributors by output, followed by India (95), Germany (71), and South Korea (65). Citation impact does not follow the same ordering: the United States accumulated the highest citation count (2,404), followed by South Korea (1,709) and Germany (1,489), indicating that productivity and influence are unevenly distributed across the field. The 10 most productive countries are listed in Table 3.

**Table 3:** Ten most productive countries in the Scopus subset (n = 855). Documents involving several countries are counted for each.

Rank	Country	Documents	Citations
1	China	163	1,299
2	United States	155	2,404
3	India	95	1,037
4	Germany	71	1,489
5	South Korea	65	1,709
6	Turkey	59	695
7	Italy	48	747
8	Brazil	40	638
9	United Kingdom	34	717
10	Saudi Arabia	33	1,119

The network resolves into six clusters with a clear regional structure. A first grouping links China, Hong Kong, and Japan; a second grouping connects the United States with South Korea, Turkey, Canada, and Denmark; a third grouping associates Germany, the Netherlands, Iran, Austria, Romania, and Poland; a fourth grouping brings together India, Iraq, Taiwan, Malaysia, and Australia; a fifth grouping links the United Kingdom, Saudi Arabia, Egypt, Sweden, and Belgium; and a Southern European cluster connects Italy, France, Spain, and Israel. The United States occupies the most central position, bridging the East Asian and European groupings. This pattern indicates that international collaboration in this field remains partly structured along regional lines, with a limited number of countries acting as connectors between otherwise separate research communities.

Clinical and technical barriers

Dataset Heterogeneity and Imaging Protocol Variability

Methodological heterogeneity is the barrier most consistently reported across the corpus. AI models are trained on highly varied imaging protocols, ranging from two-dimensional lateral cephalograms to three-dimensional cone-beam computed tomography (CBCT), which limits cross-compatibility between studies [13]. Differences in image modality, spatial resolution, and acquisition parameters, such as voxel size, field of view, and dose settings, alter the input feature distribution a model encounters during training. When a model trained on one acquisition protocol is applied to images acquired under another, this domain shift can degrade landmark or segmentation accuracy at external validation even when in-sample performance was high. Annotation protocols compound this problem, since differences in how landmarks are defined and traced between centres introduce label noise that constrains both training reliability and cross-study comparability.

Dataset Bias

Dataset bias takes several distinct forms that are frequently conflated. Single-centre bias arises when a model is trained and validated on data from one institution, so that it implicitly learns that institution's patient mix, scanner hardware, and clinical protocols rather than generalisable anatomical patterns. Demographic bias occurs when training cohorts under-represent certain ethnicities, age groups, or craniofacial phenotypes, reducing reliability for patients outside the training distribution. Spectrum bias refers to cohorts that over-represent clear-cut or severe presentations while under-representing borderline cases, inflating apparent accuracy relative to real-world case mix. Class imbalance describes a skewed distribution between outcome categories, which can bias a classifier towards the majority class. The predominance of single-centre retrospective designs in this literature makes the first of these particularly prevalent [14].

Algorithmic Interpretability

The black-box character of DL networks limits clinical interpretability and raises ethical and medicolegal difficulties [15]. This is of particular consequence in maxillofacial surgery, where errors may produce permanent sequelae, such as inferior alveolar nerve injury during mandibular osteotomies, a complication whose reported incidence varies widely across the literature and remains inconsistently assessed [16].

Generative AI and LLM-Specific Risks

Recent data have documented specific limitations of generative systems in this specialty. Jain et al. evaluated 500 ChatGPT-4-generated references across 10 standardised prompts in oral and maxillofacial surgery and found that 26.4% (132/500) were entirely fabricated, with a further 33.0% partially correct; this hallucination rate was measured for ChatGPT-4 specifically, and extending it to generative systems as a class would require comparable audits of other models [17]. A national survey by Kranc et al. of 81 oral and maxillofacial surgery residents found that 79.0% had used a large language model during training, while 97.5% reported having received no formal instruction on model limitations, bias or safety [18]. The prominence of the generative AI cluster in the present analysis should therefore be read alongside these findings.

Future strategic directions

The thematic structure identified here suggests three directions. First, the separation between the imaging clusters and the surgical planning cluster indicates that multimodal fusion architectures - integrating cone-beam computed tomography bone segmentation with intraoral scans and patient-specific soft tissue photography - remain comparatively underdeveloped relative to single-modality work. Such virtual planning frameworks have already demonstrated clinical efficiency and applicability across complex maxillofacial and mandibular reconstructions [19], which provides a practical baseline against which multimodal AI-driven extensions can be evaluated. Second, the regional structure of the co-authorship network, combined with the predominance of single-centre datasets, supports the adoption of federated learning frameworks that would allow models to be trained across institutions without transferring raw patient data. Third, the persistence of interpretability as a reported barrier suggests that explainable AI methods providing visual attribution, such as gradient-weighted class activation mapping, are needed to establish clinical trust and to preserve the operating surgeon as the accountable decision-maker.

Limitations

Several limitations should be weighed when interpreting these findings. First, the analysis is restricted to English-language records indexed in three databases. Web of Science was available to the authors only through a natural-language Smart Search interface that does not support reproducible Boolean querying, and Embase was not accessible; relevant work indexed exclusively in either database, or published in other languages, may therefore have been missed. Second, title and abstract screening relied on two AI-assisted passes with author adjudication rather than on fully independent human double screening, and the observed inter-pass agreement was moderate (Cohen's kappa: 0.33), which is why all discrepant records were individually adjudicated. Third, the country-level co-authorship analysis was restricted to the Scopus subset (n = 855), because complete affiliation metadata could not be reliably extracted from the pooled reference-manager file used for the keyword analyses; collaboration patterns should therefore be interpreted as representative of the Scopus-indexed portion of the corpus rather than of the full corpus. Fourth, keyword co-occurrence analysis depends on author-assigned and database-assigned terminology, which is inconsistent across sources; a thesaurus was applied to mitigate this, but residual terminological variation cannot be excluded. Finally, bibliometric mapping describes the structure of the published literature and cannot establish the clinical validity or safety of the methods it maps.

## Conclusions

This bibliometric mapping of 854 documents indicates that AI research in orthognathic and maxillofacial surgery is organised into five thematic clusters and has undergone a marked shift in emphasis within the published record, with generative language applications emerging as the most recent thematic front alongside established work on cephalometric automation and surgical planning. International collaboration is structured into six regional clusters, with China and the United States as principal contributors and the United States as the main connector between research communities. These are observations about the structure of the literature rather than clinical conclusions, and this study design cannot assess whether the methods described improve patient outcomes. The barriers most consistently reported across this corpus - methodological heterogeneity, dataset homogeneity, and limited algorithmic interpretability - remain to be addressed before wider clinical translation, and future work would benefit from multimodal fusion architectures, federated learning frameworks, and explainable AI methods that keep the maxillofacial surgeon at the centre of the decision-making process.
